# Assessment of the Relationship between Postpartum Health and Mid-Lactation Performance, Behavior, and Feed Efficiency in Holstein Dairy Cows

**DOI:** 10.3390/ani11051385

**Published:** 2021-05-13

**Authors:** Malia J. Martin, Kent A. Weigel, Heather M. White

**Affiliations:** Department of Animal and Dairy Sciences, University of Wisconsin-Madison, Madison, WI 53706, USA; mmartin37@wisc.edu (M.J.M.); kent.weigel@wisc.edu (K.A.W.)

**Keywords:** hyperketonemia, residual feed intake, activity, rumination

## Abstract

**Simple Summary:**

The transition to lactation is a physiologically challenging period in which dairy cows are more susceptible to postpartum health disorders. Postpartum health disorders are associated with negative outcomes related to milk production, reproduction, and profitability. Feed efficiency is another measure of performance, but its relationship with postpartum health disorders has not been explored. Cows with health disorders have differences in behavior that can be used to help identify them as being ill, but it is unknown how long the behavior remains different. The purpose of the present study was to determine the relationship between postpartum health disorders and mid-lactation performance, feed efficiency, and behaviors. Cows with health disorders had no difference in feed efficiency compared to healthy animals but had differences in behavior during the mid-lactation period. This work indicates that health disorders do not have long-lasting impacts on feed efficiency but may have long-lasting impacts on behavior.

**Abstract:**

The objective of this study was to investigate the relationships between postpartum health disorders and mid-lactation performance, feed efficiency, and sensor-derived behavioral traits. Multiparous cows (*n* = 179) were monitored for health disorders for 21 days postpartum and enrolled in a 45-day trial between 50 to 200 days in milk, wherein feed intake, milk yield and components, body weight, body condition score, and activity, lying, and feeding behaviors were recorded. Feed efficiency was measured as residual feed intake and the ratio of fat- or energy-corrected milk to dry matter intake. Cows were classified as either having hyperketonemia (HYK; *n* = 72) or not (*n* = 107) and grouped by frequency of postpartum health disorders: none (HLT; *n* = 94), one (DIS; *n* = 63), or ≥2 (DIS+; *n* = 22). Cows that were diagnosed with HYK had higher mid-lactation yields of fat- and energy-corrected milk. No differences in feed efficiency were detected between HYK or health status groups. Highly active mid-lactation time was higher in healthy animals, and rumination time was lower in ≥4th lactation cows compared with HYK or DIS and DIS+ cows. Differences in mid-lactation behaviors between HYK and health status groups may reflect the long-term impacts of health disorders. The lack of a relationship between postpartum health and mid-lactation feed efficiency indicates that health disorders do not have long-lasting impacts on feed efficiency.

## 1. Introduction

The transition to lactation is a physiologically challenging period that is characterized by metabolic, endocrine, and immunological changes, as well as a negative energy balance [1,2,3,4]. Inadequate adaptation to the negative energy balance and the onset of lactation puts cows at risk for metabolic disorders, including hyperketonemia (HYK), a displaced abomasum, and hypocalcemia [5,6,7], while immunosuppression predisposes cows to a retained placenta and infectious diseases, such as mastitis and metritis [1,8]. Transition cow health disorders are associated with negative outcomes in terms of milk production loss [9,10,11], reduced reproductive performance [6,10,12], risk of involuntary culling [13,14], and loss of profitability due to the direct and indirect costs associated with the disorder [15,16].

While the negative outcomes related to milk yield, reproduction, and profit have been extensively documented, the relationships between transition cow health disorders and feed efficiency have not been investigated thoroughly. Residual feed intake (RFI) is a measure of feed efficiency that is defined as the difference between the observed and predicted feed intakes [17]. Because RFI accounts for known energy sinks, including maintenance, body weight change, and secreted milk energy [17], cows with negative RFI values consume less feed than expected; thus, they are more efficient than their contemporaries. Sixty percent of the variation in RFI has been attributed to six biological traits: activity, feeding behavior, other behaviors, body reserve changes, rumen temperature, and digestibility [18]. It is likely that these or other yet-to-be-measured behavioral or physiological traits will be designated as additional energy sinks once their effects are more fully understood and methods for their measurement are confirmed. It is possible that metabolic disruptions that alter nutrient utilization [19] may influence RFI, especially when associated with body tissue mobilization or shifts in nutrient partitioning. Previous research has examined the association between RFI and subsequent lactation HYK [20]; however, no research has examined the impact of HYK on RFI within the same lactation. Given the potential for HYK to be associated with changes in nutrient partitioning at the tissue [21] and whole-animal levels [19], understanding this relationship is an important aspect of selecting for RFI. 

Identifying and describing additional energy sinks that contribute to variation in RFI, including those associated with health, immune function, activity, feeding behavior, or nutrient utilization, will enhance our understanding of feed efficiency. Until recently, methods to assess within- and between-cow variations in biological traits, such as activity, feeding behavior, rumination, and body temperature have been lacking. Due to the increasing availability of wearable sensors and other precision management technologies, researchers have been able to characterize lying, activity, feeding, and rumination behaviors during the transition-to-lactation period and reveal associations between these variables and postpartum health disorders [22,23]. However, these associations are limited to behaviors that are observed in the first three to four weeks of lactation. It is possible that relationships between postpartum health disorders and behavioral traits could change over the course of lactation, yet relationships between postpartum health disorders and mid-lactation behaviors have not been explored. Therefore, the objective of this study was to investigate the relationships between postpartum health disorders and mid-lactation performance, feed efficiency, and sensor-derived behavioral traits. 

## 2. Materials and Methods 

All animal protocols were approved by the University of Wisconsin-Madison College of Agricultural and Life Sciences Animal Care and Use Committee (IACUC #A005802 and #A005658). Multiparous Holstein dairy cows were monitored for postpartum health disorders for the first 21 DIM and then enrolled in feed efficiency trials for 45 days between 50 to 200 DIM. The cows were housed in a sand-bedded freestall barn at the University of Wisconsin-Madison Emmons Blaine Dairy Cattle Research Center (Arlington, WI, USA). 

### 2.1. Early Lactation Sample Collection and Analysis

From March 2019 through to November 2019, multiparous cows (*n* = 260) at the University of Wisconsin-Madison Emmons Blaine Dairy Cattle Research Center (Arlington, WI, USA) were enrolled after parturition. All multiparous cows were housed and fed together. Blood samples were taken twice weekly after the morning milking and within 30 minutes of fresh feed delivery from the coccygeal vessel of all cows between 5 and 18 DIM. Immediately after blood sampling, the blood was analyzed for BHB concentrations using the BHBCheck cow-side meter (PortaCheck, Moorestown, NJ, USA). The BHBCheck meter is a ketone body and glucose meter with a sensitivity of 91% and specificity of 93% for detecting HYK in bovine blood samples, as compared with a colorimetric laboratory assay [24]. Hyperketonemia was defined as at least one blood sample with a blood BHB ≥ 1.2 mmol/L. If HYK was suspected during the daily health monitoring (described below) on a non-sampling day, an additional blood sample was taken as part of the herd management, resulting in some cows with five samples or a positive diagnosis prior to 5 DIM. 

Cow health was assessed daily for the first 10 DIM as part of standard fresh cow management. Daily health monitoring included visual observation (overall physical appearance and attitude, respiration rate, rumination activity, presence of uterine discharge), rectal temperature, and urine ketones using a semiquantitative dipstick. After 10 DIM, the cows were monitored visually daily, with rectal temperatures or further monitoring limited to cows that were suspected of having health disorders. Additionally, milk production was monitored daily, and deviations from the expected milk production were used to identify cows with health disorders throughout lactation. Diagnosis and treatment of health disorders were conducted and documented according to standard operating procedures by trained farm personnel with veterinary supervision. Briefly, monitored health disorders included (1) HYK: blood BHB ≥ 1.2 mmol/L; (2) milk fever: off-feed, cold ears, muscle tremors, inability to rise, or lying in a lateral recumbency; (3) retained placenta: failure regarding placenta expulsion within 12 hours after calving; (4) metritis: fever and foul-smelling uterine discharge; (5) mastitis: milk with an abnormal appearance (presence of flakes or clots), swelling or pain in the udder, sometimes with systemic sickness (off-feed, lethargy, diarrhea, dehydration); (6) displaced abomasum: off-feed with an audible “ping” sound when tapping over the left or right abdominal cavity.

Health events were recorded for the first 21 DIM and cows were categorized into three health status groups based on the presence of health disorders: healthy cows that were not diagnosed with any health disorder (HLT), cows that were diagnosed with one health disorder (DIS), and cows diagnosed with two or more health disorders (DIS+). To investigate HYK specifically, the cows were also grouped by HYK status: at least one blood BHB sample ≥ 1.2 mmol/L (HYK) or all samples below 1.2 mmol/L (nonHYK).

### 2.2. Mid-Lactation Sample Collection and Analysis

From June 2019 through to February 2020, multiparous cows, including some of those sampled in the postpartum period (*n* = 205), between 50 and 200 DIM were enrolled in one of four feed efficiency trials of 45 days in duration. All cows within a trial were fed an identical TMR diet that met or exceeded the requirements of a 650 kg lactating cow based on the NRC (2001). The TMR was mixed once daily at 1100 h and distributed twice daily at 1100 h and 1500 h into 32 electronic roughage intake control bins (Hokofarm Group, Marknesse, The Netherlands). Cows had ad libitum access to water and feed from all bins. Daily intakes were recorded electronically using the electronic roughage intake control bins. Refusals targeted 10% and were emptied daily before morning feeding. 

Samples of individual feedstuffs and TMR were collected weekly. Weekly TMR and individual feed samples were dried for 24 h in a 105 °C forced-air oven to determine the DM mass. Weekly samples of individual feeds were dried at 55 °C in a forced-air oven for 48 h, ground to pass a 1 mm screen (Wiley Mill, Arthur H. Thomas, Philadelphia, PA, USA), and composited across each trial for nutrient composition analysis. Individual composited feeds were analyzed at a commercial lab (Dairyland Laboratories, Inc., Arcadia, WI, USA), as described previously [25]. Diet composition and nutrient analysis are presented in Supplemental Table S1. 

Cows were milked twice daily at 0400 h and 1500 h. Milk yields were recorded electronically at each milking, and milk samples were taken weekly at four consecutive afternoon and morning milkings and analyzed for milk composition at a commercial laboratory (AgSource, Menominee, WI, USA), as described previously [25]. Milk samples with a composition of more than double or less than half of the next highest or lowest value for that cow, respectively, were considered biologically implausible and removed (milk fat, *n* = 13). Component yields for specific milking sessions were calculated by multiplying the milk yield by the milk composition for that milking session, and daily yields of the components were calculated by summing successive afternoon and morning milkings. The secreted milk energy was determined as in NRC (2001; Equation 2-15): [(9.29 × milk fat (kg)) + (5.63 × true protein (kg)) + (3.95 × lactose (kg)], using the coefficient for true protein instead of crude protein. Similarly, 3.5% fat-corrected milk (FCM) was calculated as [(0.4324 × milk yield (kg)) + (16.216 × milk fat (kg))], and energy-corrected milk (ECM) was calculated as [(0.327 × milk yield (kg)) + (12.95 × milk fat (kg)) + (7.65 × milk protein (kg))] [26]. 

Body weights were recorded for three consecutive days at the beginning (wk 1), middle (wk 4), and end (wk 7) of each trial. Body weights with a coefficient of variation greater than 5% within a week had the observation deleted, such that when removed, it minimized the coefficient of variation (n = 17). The daily body weight change was calculated by applying the LINEST function in Microsoft Excel (Microsoft Corp., Redmond, WA, USA) to the body weight measurements taken over time. Lastly, BCS was independently assessed by three trained individuals during the same weeks as the body weight measurements using a five-point scale with quarter-point increments [27]. 

Cow sensor data were collected via SMARTBOW (Zoetis, Kalamazoo, MI, USA) ear-tag accelerometers fitted to individual animals. This system utilizes proprietary algorithms to generate location and behavior measures that have been described and validated previously [28,29,30]. Behavioral data included rumination time, lying time, and activity categorized as inactive, active, and highly active. Precise definitions of these behaviors are protected as intellectual property. Localization data were also recorded, and times spent in different locations within the barn were categorized into areas, such as the pen, parlor, and feedbunk. Data were collected continuously and summarized by hour and day. Behavioral data (activity, rumination, lying) that were missing one (1.95%) or two (0.05%) hours within a day were imputed using the mean of that hour for the same cow on the four previous and four subsequent days when computing the daily totals. Localization data that were missing more than one hour per day (9%) were deleted and treated as missing. 

### 2.3. RFI Calculation

Residual feed intake was determined by computing the dry matter intake (DMI) as a function of major energy sinks using R (version 4.0.1; R Core Team, 2020). The RFI model was applied to each of the feed efficiency trials separately, recognizing that energy sink relationships could vary across trials [31]. The RFI model was:

DMI_i_ = μ + β_1_ × MilkE_i_ + β_2_ × MBW_i_ + β_3_ × ΔBW_i_ + β_4_ × DIM_i_ + RFI_i_, (1)

where DMI_i_ was the observed average DMI (kg/day) of the ith cow and μ was the overall mean. MilkE_i_, MBW_i_, ΔBW_i_, and DIM_i_ were the secreted milk energy, metabolic body weight (body weight^0.75^), daily change in body weight, and midpoint days in milk, respectively, for the ith cow. Regression coefficients β1, β2, β3, and β4 corresponded to the secreted milk energy, metabolic body weight, change in body weight, and midpoint days in milk, respectively. The random residual, RFI_i_, represented the RFI of the ith cow. 

### 2.4. Statistical Analysis

Mid-lactation performance and intake data were averaged across the study period. In total, 179 cows had complete records from the early- and mid-lactation sampling periods for inclusion in the analysis of production, feed intake, and feed efficiency, whereas a subset of 169 cows had complete sensor-derived behavioral data for inclusion in the behavioral analyses. The following statistical model was used for the analysis of the relationships between early postpartum HYK status and mid-lactation performance and behavior:

y_i_ = μ + HYK_i_ + Parity_i_ + Trial_i_ + β_1_ DIM_i_ + β_2_ MEMilk_i_ + e_i_ (2)

where y_i_ was the mid-lactation phenotype of cow i for DMI, BCS, body weight, milk yield, FCM, ECM, fat yield, fat percentage, protein yield, protein percentage, RFI, FCM/DMI, ECM/DMI, lying time, rumination time, total active time, active time, highly active time, or time spent at the feedbunk; HYK_i_ was the fixed effect of early postpartum hyperketonemia status of cow i, with two levels corresponding to HYK or nonHYK; Parity_i_ was the fixed effect of lactation number of cow i, with three levels corresponding to lactation 2, 3, or ≥4; Trial_i_ refers to the fixed effect of the feed efficiency trial in which cow i participated, with four levels; DIM_i_ referred to days in milk of cow i at the midpoint of her feed efficiency trial, with regression coefficient β_1_; MEMilki was the previous lactation mature-equivalent 305-day milk yield of cow i, with regression coefficient β_2_; e_i_ was the random residual. The model for assessing the relationship between early postpartum health events and mid-lactation performance and behavior was identical to the model shown above, with the exception that HYK_i_ was replaced with Health_i_, which represented the early postpartum health status of cow i, with three levels corresponding to HLT, DIS, and DIS+. All statistical analyses were performed in R (version 4.0.1; R Core Team); significance was declared at *p* ≤ 0.05 and a tendency was declared at 0.05 < *p* ≤ 0.10. Least-square means were separated using Tukey’s studentized adjustment when *p* ≤ 0.05, where appropriate. Interaction terms were retained in the model when significant at *p* ≤ 0.05. When residuals were not normal, a Box–Cox transformation was performed, and the values are reported as back-transformed means and 95% confidence intervals. 

## 3. Results and Discussion

### 3.1. Hyperketonemia Descriptive Statistics

In total, 260 cows were sampled during the early lactation period, and 108 (41.5%) were diagnosed with HYK. The average DIM and BHB concentrations at the first positive test were (mean ± SD) 9.6 ± 4.8 days and 1.9 ± 1.0 mmol/L, respectively. Of these, 179 cows had complete mid-lactation records for milk production, milk composition, and feed intake variables, and were retained for analysis. The incidence of HYK in the merged dataset was 40.2% (HYK, *n* = 72; nonHYK, *n* = 107), with an average DIM at the first test of 9.3 ± 4.7 and average BHB concentration at the first test of 1.8 ± 0.7 mmol/L (Table 1), indicating negligible evidence of selection bias based on HYK status. Incidence of HYK and frequency of health disorders by lactation number for the merged dataset are reported in Table 1 and Table 2, respectively. Herd-level incidence of HYK has been reported to range from 20 to 60%, spanning the range observed in this study [20,32,33]. However, this study did not include primiparous cows, which typically have lower HYK incidence [32], and this may have caused a higher incidence rate in the current study. Incidence within parity has been reported as 17 to 37% for second lactation cows and 26 to 61% for third and greater lactation cows, which are also consistent with the present study [20,33,34]. 

### 3.2. Association of HYK Diagnosis with Mid-lactation Performance and Efficiency

Midpoint DIM of the mid-lactation data collection period were (mean ± SD) 111 ± 23 and 113 ± 27 days for HYK and nonHYK cows, respectively. Cows diagnosed with HYK in the postpartum period had similar body weights at mid-lactation as nonHYK cows (*p* = 0.14; Table 3); however, HYK cows had a lower BCS in mid-lactation (*p* = 0.05) compared with nonHYK cows (Table 3). Although precalving and early lactation body weight and BCS were not available in the current study, previous research has indicated cows with a higher precalving BCS, as well as those that lost more BCS during the transition period, had a greater risk of developing HYK [20,32,35]. 

Cows that were diagnosed with HYK tended to produce more milk (*p* = 0.06; Table 3) than nonHYK cows in mid-lactation. Generally, HYK is associated with a reduced milk yield in the first 30 DIM [14,36], although some studies have reported an increase [20,35]. Hostens et al. [11] modeled lactation curves for healthy cows, cows with HYK and no other health disorder (uncomplicated HYK), and cows with HYK and another health disorder (complicated HYK) and reported that cows with complicated or uncomplicated HYK had a slower increase in milk production and a delayed peak of lactation compared with nonHYK cows. Cows with complicated HYK had lower peak milk and cumulative 305-day milk yield than healthy cows and those with uncomplicated HYK. However, for cows with uncomplicated HYK, there was no difference in peak milk yield or cumulative 305-day milk yield, indicating that HYK alone did not alter full lactation performance. Likewise, cows that were predicted to have HYK through a multiple linear regression analysis of milk and performance variables but not culled within 60 days postpartum had similar peak milk yield to those that were not predicted to have HYK [19].

Cows that were diagnosed with HYK produced more FCM and ECM (*p* ≤ 0.03; Table 3) than nonHYK cows in mid-lactation. The increase FCM and ECM result from an increase in milk fat yield (*p* = 0.03; Table 3), as milk protein concentration and yield were similar between groups (*p* ≥ 0.14; Table 3). Interestingly, for milk fat concentration, there was a significant interaction of HYK diagnosis and previous lactation ME305 (*p* = 0.02), wherein cows diagnosed with HYK had lower milk fat concentrations than their nonHYK contemporaries among cows with a low previous lactation ME305, but higher milk fat concentrations than their nonHYK contemporaries among cows with a high previous lactation ME305. Greater milk fat and lower milk protein concentrations in the first 30 DIM were observed for cows diagnosed with HYK [20,32,35,36]. Higher yields of milk fat, FCM, and ECM mid-lactation for HYK cows in the present study were consistent with the findings of Rathbun et al. [20] in the first 30 DIM. Energy-corrected milk at the first test was also greater in cows that were predicted to have HYK in a multiple linear regression analysis of milk and performance variables in 240,000 lactation records [19]. 

**Figure 1:**
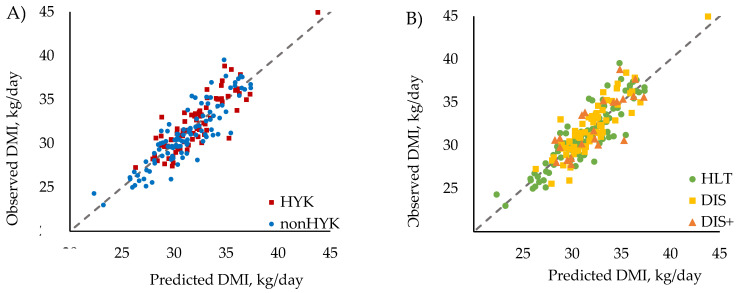
Observed versus predicted plots of mid-lactation DMI (R^2^ = 0.78; line of unity = gray dashed line), which was plotted for cows diagnosed with or without hyperketonemia (HYK or nonHYK, respectively) within the first 18 days in milk (panel **A**) and for cows diagnosed with none, one, or two or more postpartum health disorders (HLT, DIS, and DIS+, respectively) within the first 21 days in milk (panel **B**). Data points above the line of unity represent cows that consumed more feed than predicted and were less feed efficient (positive RFI). Data points below the line of unity represent cows that consumed less feed than predicted and were more feed efficient (negative RFI).

Although cows diagnosed with HYK had higher mid-lactation FCM and ECM and similar DMI to nonHYK cows, no difference was detected in any measure of feed efficiency (*p* ≥ 0.12; Table 3, Figure 1A). Residual feed intake is the unexplained variation in feed intake after accounting for metabolic body weight, secreted milk energy, body weight change, and days in milk. Research groups have investigated other potential energy sinks, including differences in feeding behavior and digestibility [18], but altered metabolism could also contribute to variation in RFI. It is possible that HYK, which is characterized by altered metabolism, could have a long-term impact on feed efficiency. Given the lack of relationship between RFI and HYK diagnosis in this study, it appears that postpartum HYK did not contribute to mid-lactation variation in RFI, suggesting that the use of RFI as a selection tool will not inadvertently lead to the selection of animals that are predisposed to greater risk of HYK. Rathbun et al. [20] also investigated the relationship between RFI and subsequent lactation HYK by grouping cows into quartiles based on the phenotypic RFI in a previous lactation and found no relationship between RFI quartile and maximum postpartum BHB concentration in the subsequent lactations. Substantiating the findings of Rathbun et al. [20] in the current study is warranted given that the repeatability of RFI across lactations is low to moderate [31,37]. Furthermore, the presence of HYK or other health disorders shifts DMI and nutrient use, which could have sequential effects as cows progress into lactation. Investigating the relationship between same lactation HYK as a contributor to RFI should be conducted by utilizing a larger dataset to confirm the findings presented herein. Given the intensity of measuring blood BHB and RFI, it is challenging to generate large datasets of this type; however, there may be a role for using proxy measures for feed intake [38,39] and predicted HYK status [40,41,42] to expand the exploration of these relationships.

### 3.3. Association of Health Status with Mid-lactation Performance and Efficiency

Of the 179 cows retained for mid-lactation analysis, 94 were categorized as HLT, 63 were categorized as DIS, and 22 were categorized as DIS+. The midpoint DIM during the mid-lactation data collection period were (mean ± SD) 112 ± 26, 111 ± 25, and 114 ± 25 days for HLT, DIS, and DIS+, respectively. Across the three health status groups, there were no differences in any of the measures of feed efficiency (*p* ≥ 0.40; Table 4, Figure 1B), nor were differences detected in mid-lactation DMI, milk yield, or milk composition (*p* ≥ 0.18; Table 4). Further, mid-lactation BCS and body weight did not differ (*p* ≥ 0.29; Table 4) by postpartum health status. The lack of relationship between feed efficiency and health status observed in the present study is supported by Nehme Marinho et al. [43], who found no association between RFI quartile and postpartum health disorders (excluding HYK, which was not evaluated). Hostens et al. [11] modeled lactation curves for cows that were grouped into similar HLT, DIS, and DIS+ categories and reported that the lactation curves were altered by health status. Specifically, the incline in milk production was slower and the peak milk yield was delayed, but the persistency was greater for DIS and DIS+ cows as compared with HLT cows. Despite the differences in the lactation curves, DIS cows had a similar peak milk yield and a 305-day cumulative milk yield as the HLT cows. However, DIS+ cows had lower peak milk yield and 305-day cumulative milk yield than the HLT or DIS cows. Given the low frequencies of health disorders observed in the present study, excluding HYK, it was possible that this study lacked the power to detect differences by health status, particularly for DIS+. 

### 3.4. Association of HYK Diagnosis with Mid-lactation Behavior

No differences in the total active time, active time, or time spent lying during the mid-lactation period were detected between nonHYK and HYK cows (*p* ≥ 0.14; Table 5). Previously, cows diagnosed with HYK were shown to be less active compared with nonHYK cows during the first three weeks of lactation [22,44] and in the days before and immediately after diagnosis [45]. Itle et al. [46] observed longer standing times in the week prior to calving, but no difference in standing times after calving for cows that would develop prolonged hyperketonemia with a BHB concentration ≥ 3.0 mmol/L. Conversely, while no differences in lying time prepartum were detected, multiparous HYK cows spent more time lying in the first two [47] and four weeks of lactation [48]. Any impact of HYK on lying and standing behaviors in the transition period appeared to be diminished by the mid-lactation period, as the lying times of HYK and nonHYK cows were similar in the present study. Cows diagnosed with HYK in this study spent less time being highly active (*p* = 0.02) during mid-lactation compared with their nonHYK counterparts (Table 5). Time spent being highly active is a sensor-specific measure that is used to generate an estrus alert; however, cows can spend time being highly active without being in estrus. Rutherford et al. [49] investigated the activity around estrus between HYK and nonHYK and found cows with HYK had shorter duration and lower peak activity at first estrus and first insemination than nonHYK cows. While we were unable to attribute the source of the difference in high activity to specific behaviors, this finding could reflect differences in behavior that are remnants of postpartum HYK. 

**Figure 2:**
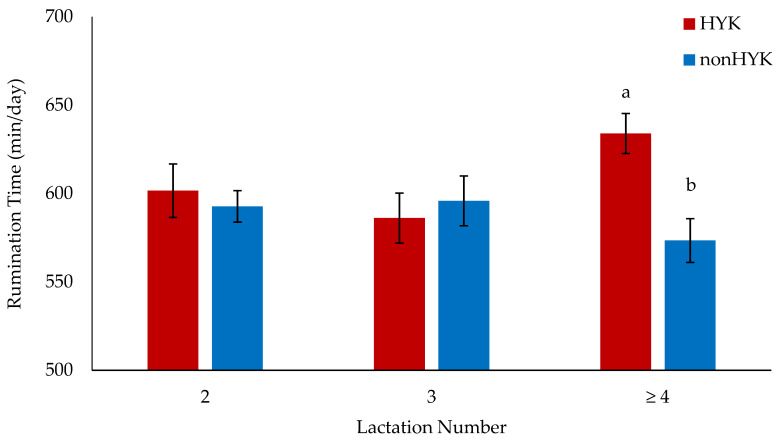
Effect of the interaction between lactation number and diagnosis on the effect of rumination time (*p* = 0.02) measured between 50 and 200 days in milk for cows diagnosed with or without hyperketonemia (HYK or nonHYK, respectively) within the first 18 days in milk. Differences within a given lactation number are denoted with superscripts. For nonHYK, *n* = 54, 24, and 29 for lactation numbers 2, 3, and ≥4, respectively. For HYK, *n* = 18, 21, and 33 for lactation numbers 2, 3, and ≥4, respectively.

There was a significant interaction of HYK diagnosis and lactation number on the effect of rumination time in mid-lactation, wherein there was no difference in rumination time between HYK and nonHYK cows in their second or third lactation, but fourth lactation and greater cows that were diagnosed with HYK ruminated more than their nonHYK counterparts (*p* = 0.02; Figure 2). Although previous research has reported no relationship between age and rumination time [50], there is evidence for a relationship between HYK diagnosis and mid-lactation rumination time. Rumination time in the first 7 weeks of lactation is negatively associated with blood BHB concentrations [51]. In a study utilizing primiparous and multiparous cows, rumination time decreased in cows with HYK in the first week of lactation [22]. Kaufman et al. [52] analyzed rumination separately for primiparous and multiparous cows and found that, while no differences were observed in primiparous cows, multiparous cows with HYK and no other disorder tended to ruminate less in the weeks before and after calving, while cows with HYK and at least one other disorder had lower rumination times in the week before and two weeks after calving as compared with nonHYK cows. Unfortunately, no further distinction between multiparous cows was made in that study; therefore, differences in the rumination time between the second, third, and fourth or greater lactation cows were unavailable. The findings in the present study indicated a positive relationship between HYK and mid-lactation rumination time, while research conducted during the transition period concluded a negative relationship between rumination time and HYK. These differences may indicate that the relationship between HYK status and rumination time change over time and warrants further investigation.

Time at the feedbunk was approximated based on the location of the cow and may give an indication of the eating time. No difference was observed (*p =* 0.46; Table 5) in time spent at the feedbunk during mid-lactation between HYK and nonHYK cows in the present study. Schirmann et al. [53] investigated rumination and feeding behaviors of HYK and nonHYK cows in the week before and three weeks after calving, and while no difference in rumination time was detected, the authors found that cows that developed HYK spent less time eating in the week before and two weeks following calving; this difference disappeared by the third week of lactation. Similarly, Goldhawk et al. [54] observed that HYK cows spent less time at the feedbunk in the two weeks before and the first week after calving than their nonHYK contemporaries, but the times were similar during the second and third weeks of lactation. Both studies reported a reduced DMI during the weeks that time at the feedbunk was reduced, indicating agreement between time at the feedbunk and the consumed DMI. Similar DMI (*p* = 0.35; Table 3) between the HYK and nonHYK cows in the current study supports the lack of difference in time at the feedbunk.

### 3.5. Association of Health Status with Mid-lactation Behavior

Among the three health status groups, no differences were detected in the total active time, active time, or time spent lying (*p* ≥ 0.37; Table 6). Cow activity was reported to be reduced in the three weeks before and three weeks after calving, as well as near the time of diagnosis for cows with health disorders, but the severity and timing of the reduction in activity were specific to each disorder [22,45,55,56]. Neave et al. [57] reported longer lying times in the two weeks before calving, as well as longer duration of lying bouts without differences in total lying time in the week after calving, for cows diagnosed with metritis. Other studies indicated longer lying times in the week after calving for cows diagnosed with health disorders [58,59], and Stevenson et al. [60] reported greater resting times from two weeks before through three weeks after calving for cows with health disorders. These inconsistencies in the timing of altered behaviors could partly be due to differences in the corresponding health disorders experienced, as well as the severity and length of the duration. Additionally, differences in behaviors could be affected by trait measurement, as Tsai et al. [44] reported significant differences in lying, rumination, and activity behaviors between healthy cows and cows with health disorders with some sensors, while other sensors did not detect differences. While mid-lactation total active time and lying time were unaffected by health status in the present study, DIS cows spent less time being highly active (*p*= 0.01; Table 6) than their HLT contemporaries. Recent work using the same sensor system as the present study found that highly active time was lower in the week before and week after calving for cows that would develop health disorders relative to healthy cows, indicating this metric is capturing true differences in behavior outside of estrus behavior [59]. The current work aligns with the findings of Gusterer et al. [59] in which highly active behavior was reduced in cows with health disorders. Although highly active time is sensor-specific, it is possible that the reduction in time spent being highly active is a long-term impact of postpartum health disorders. 

**Figure 3:**
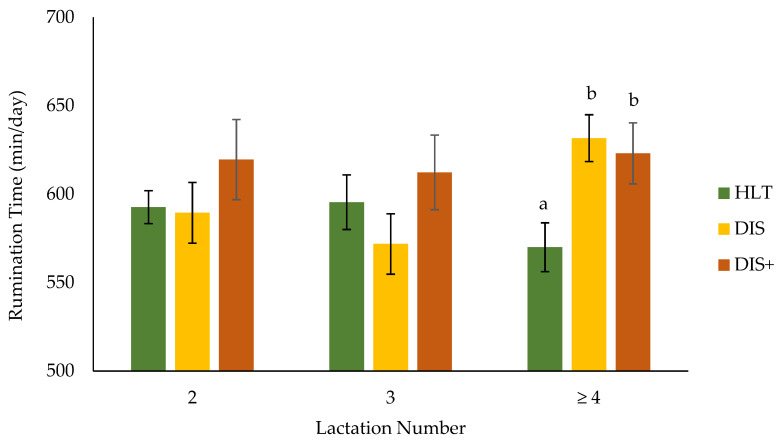
Effect of the interaction between lactation number and health status on the rumination time (*p* = 0.04) measured between 50 and 200 days in milk for cows diagnosed with none, one, or two or more postpartum health disorders (HLT, DIS, and DIS+, respectively) within the first 21 days in milk. Differences within the lactation number are denoted with superscripts. For HLT, *n* = 49, 21, and 24 for lactation numbers 2, 3, and ≥ 4, respectively. For DIS, *n* = 18, 18, and 27 for lactation numbers 2, 3, and ≥4, respectively. For DIS+, *n* = 5, 6, and 11 for lactation numbers 2, 3, and ≥4, respectively.

As observed in the HYK diagnosis groups, there was a significant interaction between health status and lactation number on the effect of rumination time during mid-lactation (*p* = 0.04; Figure 3). Cows in their second and third lactations had similar mid-lactation rumination times, regardless of their health status, whereas HLT cows in their fourth or greater lactation ruminated less than their DIS and DIS+ cow contemporaries. In a previous study, rumination time was unaltered prepartum but was reduced in the first week of lactation for cows experiencing metritis, hyperketonemia, and retained placenta, but not hypocalcemia, compared with healthy cows [22]. In another study, cows that experienced at least one health disorder had similar rumination times prepartum, but lower rumination times in the first three weeks postpartum, compared with their healthy contemporaries [60]. Both studies utilized primiparous and multiparous animals and noted differences in rumination behavior between parity groups. In another study that used only multiparous cows, the rumination time did not differ in the two weeks before and one week after calving between healthy animals and those with health disorders, but the rumination times were lower in the days before and after diagnosis for those with health disorders [59]. These studies did not classify lactation number beyond primiparous and multiparous; therefore, differences in rumination time during the transition period between second, third, and fourth or greater lactation cows were not available. Taken together, these transition cow studies indicate that the reduction in rumination time postpartum observed in cows with health disorders may depend on the health disorder, parity, and time of diagnosis. As observed with the HYK diagnosis groups, the positive relationship between health disorders and mid-lactation rumination time observed in the present study was opposite to what was observed during the transition period, indicating that long-term impacts of health disorders on rumination time should be explored further. 

No difference was observed in time at the feedbunk between the health status groups in the present study (*p* = 0.58; Table 6). During the transition period, a previous study showed that multiparous cows that subsequently developed health disorders spent less time eating in the week prior to calving and the first one to three weeks after calving than their healthy contemporaries, depending on the health disorder [53]. In that study, the reduction in eating time paralleled reductions in the DMI. A similar DMI (*p* = 0.92; Table 4) between health status groups observed in the present study supports the lack of observed difference in the time at the feedbunk.

## 4. Conclusions

Postpartum health disorders are generally associated with negative outcomes on reproduction and profitability, although the impacts on behavior and feed efficiency are not fully understood. Differences in mid-lactation behaviors by postpartum health status indicate that health disorders may have long-term impacts on behavior. The relationship between rumination time and health status was negative during the transition period and positive during mid-lactation, indicating that the relationship between rumination time and health status may change over time and warrants further investigation. Future research should investigate the relationships between behavior and health disorders using larger datasets with comprehensive health information and complete behavioral data from calving through mid-lactation. The lack of a relationship between the health status postpartum and mid-lactation RFI indicates that health disorders did not have long-lasting impacts on feed efficiency.

## Figures and Tables

**Table 1:** Descriptive statistics of the incidence and time of onset of hyperketonemia by lactation number for cows tested between 1 and 18 days in milk ^1^.

			BHB Concentration (mmol/L) at Diagnosis		Days in Milk at Diagnosis	
Lactation Number	*n*	Incidence	Mean	SD	Mean	SD
2	72	25.0%	1.6	0.5	9.4	4.7
3	45	46.7%	1.9	0.9	9.7	5.5
≥4	62	53.2%	1.8	0.6	8.9	4.4

^1^ Blood samples were collected twice weekly from each cow after morning milking and quantified using a cowside meter. A diagnosis of hyperketonemia was defined as a blood BHB ≥ 1.2 mmol/L. BHB = β-hydroxybutyrate.

**Table 2:** Percentage of cows experiencing health disorders by lactation number within the first 21 days in milk ^1,2^.

Lactation Number	Hyperketonemia	RP ^3^	Metritis	Milk Fever	DA ^4^	Mastitis
2	25% (18/72)	7% (5/72)	6% (4/72)	0% (0/72)	3% (2/72)	1% (1/72)
3	47% (21/45)	7% (3/45)	0% (0/45)	0% (0/45)	11% (5/45)	7% (3/45)
≥4	53% (33/45)	8% (5/62)	10% (6/62)	6% (4/62)	3% (2/62)	6% (4/62)

^1^ Health disorders were diagnosed by trained farm staff using protocols for each disorder. ^2^ Numbers inside the parentheses indicate the number of cases and the total number of animals within each lactation group, respectively. ^3^ Retained placenta. ^4^ Displaced abomasum.

**Table 3:** Least-squares means of performance and feed efficiency variables measured between 50 and 200 days in milk for cows diagnosed with or without hyperketonemia (HYK or nonHYK, respectively) within the first 18 days in milk.

	Diagnosis ^1^			
Item ^2^	nonHYK	HYK	SEM	*p*-Value
DMI, kg/day	31.6	32.0	0.4	0.35
BCS	3.19	3.11	0.03	0.05
Body weight, kg	747	734	7	0.14
Milk yield, kg/day	50.4	51.9	0.6	0.06
FCM, kg/day	50.2	52.2	0.6	0.01
ECM, kg/day	51.0	52.6	0.6	0.03
Milk fat concentration ^3^, %	3.50	3.55	0.06	0.03
Milk fat yield, kg/day	1.75	1.83	0.03	0.03
Milk protein concentration, %	3.06	3.01	0.02	0.14
Milk protein yield, kg/day	1.54	1.56	0.02	0.53
RFI, kg/day	−0.18	0.22	0.20	0.13
FCM/DMI	1.58	1.62	0.02	0.12
ECM/DMI	1.65	1.68	0.02	0.27

^1^ Diagnosis of hyperketonemia was defined as a blood BHB ≥ 1.2 mmol/L between 1 and 18 days in milk. ^2^ DMI = dry matter intake; BCS = body condition score; FCM = fat-corrected milk; ECM = energy-corrected milk; RFI = residual feed intake. ^3^ Significant interaction of diagnosis and previous lactation ME305 (*p* = 0.02).

**Table 4:** Least-squares means of the performance and feed efficiency variables that were measured between 50 and 200 days in milk for cows diagnosed with none, one, or two or more health disorders (HLT, DIS, and DIS+, respectively) within the first 21 days in milk.

	Health Status ^1^				
Item ^2^	HLT	DIS	DIS+	SEM	*p*-Value
DMI, kg/day	31.7	31.8	31.9	0.6	0.92
BCS	3.18	3.13	3.16	0.05	0.46
Body weight, kg	747	732	747	13	0.29
Milk yield, kg/day	51.0	51.3	50.2	1.1	0.70
FCM, kg/day	50.5	51.9	50.4	1.1	0.21
ECM, kg/day	51.3	52.3	51.0	1.1	0.37
Milk fat concentration, %	3.48	3.59	3.52	0.11	0.40
Milk fat yield, kg/day	1.75	1.83	1.76	0.05	0.18
Milk protein concentration, %	3.06	3.02	3.02	0.04	0.47
Milk protein yield, kg/day	1.56	1.54	1.52	0.04	0.67
RFI, kg/day	−0.17	0.16	0.13	0.36	0.46
FCM/DMI	1.59	1.62	1.58	0.03	0.40
ECM/DMI	1.66	1.68	1.64	0.03	0.56

^1^ Health status was defined as either none, one, or two or more postpartum health disorders, including hyperketonemia, retained placenta, metritis, milk fever, displaced abomasum, and mastitis. ^2^ DMI = dry matter intake; BCS = body condition score; FCM = fat-corrected milk; ECM = energy-corrected milk; RFI = residual feed intake.

**Table 5:** Least-squares means and 95% confidence intervals of the behavioral variables measured between 50 and 200 days in milk for cows diagnosed with or without hyperketonemia (HYK or nonHYK, respectively) within the first 18 days in milk.

	Diagnosis ^1^		
Behavior, min/Day	nonHYK	HYK	*p*-Value
Lie	809 [788, 831]	808 [782, 834]	0.92
Ruminate ^2^	587 [574, 601]	607 [591, 623]	0.61
Total active	1034 [1021, 1047]	1035 [1020, 1050]	0.93
Active	958 [943, 973]	975 [957, 992]	0.14
Highly active ^3^	48 [38, 61]	32 [24, 41]	0.02
Feedbunk	221 [214, 228]	225 [217, 233]	0.46

^1^ Diagnosis of hyperketonemia was defined as a blood BHB ≥ 1.2 mmol/L between 1 and 18 days in milk. ^2^ Significant interaction of diagnosis and lactation number (*p* = 0.02). ^3^ Log-transformed, back-transformed values are shown.

**Table 6:** Least-squares means and 95% confidence intervals of behavioral variables that were measured between 50 and 200 days in milk for cows diagnosed with none, one, or two or more health disorders (HLT, DIS, and DIS+, respectively) within the first 21 days in milk.

	Health Status ^1^			
Behavior, min/Day	HLT	DIS	DIS+	*p*-Value
Lie	808 [784, 832]	809 [779, 839]	810 [772, 848]	1.00
Ruminate ^2^	586 [571, 601]	598 [579, 616]	618 [595, 642]	0.51
Total active	1036 [1022, 1050]	1033 [1016, 1050]	1031 [1009, 1053]	0.93
Active	957 [941, 974]	976 [956, 996]	967 [941, 993]	0.37
Highly active ^3^	52 ^a^ [44, 66]	28 ^b^ [21, 38]	37 ^a,b^ [25, 56]	0.01
Feedbunk	221 [213, 228]	223 [214, 233]	228 [216, 240]	0.58

^a,b^ Means without common letters differed significantly (*p* ≤ 0.05). ^1^ Health status was defined as either none, one, or two or more postpartum health disorders, including hyperketonemia, retained placenta, metritis, milk fever, displaced abomasum, and mastitis. ^2^ Significant interaction between the health status and lactation number (*p* = 0.04). ^3^ Log-transformed, back-transformed values are shown.

## Data Availability

Feed efficiency data were collected under a specific collaborative agreement to provide data for national genetic evaluations of U.S. dairy cattle for Residual Feed Intake and Feed Saved. These data are available on request of the corresponding author and approval by the collaborators, assuming that the purpose is not to duplicate the objectives of the national collaborative project. SMARTBOW data is subject to 3rd party data restrictions. Data was obtained from Zoetis, Inc. and can be made available by the corresponding author with the permission of Zoetis, Inc.

## Supplementary Materials

The following are available online at https://doi.org/10.5281/zenodo.4648048, Table S1: Calculated ingredient composition and nutrient analysis of the mid-lactation diets.
